# Isolation, Identification and Cytotoxicity of a New Noroleanane-Type Triterpene Saponin from *Salicornia bigelovii* Torr.

**DOI:** 10.3390/molecules20046419

**Published:** 2015-04-10

**Authors:** Fuqin Guan, Qizhi Wang, Ming Wang, Yu Shan, Yu Chen, Min Yin, Youyi Zhao, Xu Feng, Fei Liu, Jianhua Zhang

**Affiliations:** 1 College of Life Science, Nanjing Agricultural University, Nanjing 210095, China; E-Mail: tube1031aaa@126.com; 2 Jiangsu Provincial Key Laboratory for Coastal Wetland Bioresources, Institute of Botany, Jiangsu Province and Chinese Academy of Sciences/Nanjing Botanical Garden Mem. Sun Yat-Sen, Nanjing 210014, China; E-Mails: wangqizhi2003@126.com (Q.W.); wangmingwm0208@sina.com (M.W.); shanyu79@126.com (Y.S.); chenyu.1007@163.com (Y.C.); epmin@sohu.com (M.Y.); Zyyc163@163.com (Y.Z.); liufeiseu@163.com (F.L.); zjh13805171132@126.com (J.Z.)

**Keywords:** *Salicornia bigelovii* Torr., nortriterpenoid saponins, cytotoxicity, MCF-7 human breast cancer cells, apoptosis

## Abstract

*Salicornia bigelovii* Torr. has been consumed not only as a popular kind of vegetable, but also as a medicinal plant to treat hypertension, cephalalgia, scurvy and cancer. The present study was designed to investigate its chemical components and cytotoxic activity. A new noroleanane-type triterpene saponin, bigelovii C (**1**), was separated and purified from *Salicornia bigelovii* Torr., along with four known triterpene saponins **2**–**5**. The structure of bigelovii C was elucidated as 3-*O*-(6-*O*-butyl ester)-β-d-glucuropyranosyl-23-aldehyde-30-norolean-12, 20 (29)-dien-28-oic acid-28-*O*-β-d-glucopyranoside, according to various spectroscopic analysis and chemical characteristics. Besides Compounds **3** and **5**, bigelovii C had potent cytotoxicity against three human cancer cell lines, MCF7 (breast cancer), Lovo (colon cancer) and LN229 (glioblastoma), especially MCF7. Bigelovii C inhibited the growth of MCF7 cells in dose- and time-dependent manners. Flow cytometry analysis revealed that the percentage of apoptotic cells significantly increased upon bigelovii C treatment. Rh123 staining assay indicated that bigelovii C reduced the mitochondrial membrane potential. The mechanism of cell death by bigelovii C may be attributed to the downregulation of Bcl-2 and upregulation of Bax, cleaved caspase-9, caspase-7 and PARP. These results suggested that bigelovii C may impart health benefits when consumed and should be regarded as a potential chemopreventative agent for cancer.

## 1. Introduction

*Salicornia bigelovii* Torr. is a genus of annual, apparently leafless, halophytic herbs that have articulated succulent stems. Since this plant is an excellent source of minerals, protein, beta-carotene and vitamin C [1], it has been eaten in various ways, such as salads, fermented food and a seasoned vegetable [2]. In addition, *S. bigelovii* is a valuable oilseed crop for subtropical coastal deserts [3]. Interestingly, glasswort has also been used to treat hypertension, cephalalgia, scurvy and cancer [4]. Recently, the consumption of this plant as functional food and medicinal plant has increased significantly because of its beneficial effects.

A few chemical constituents were reported from *Salicornia* plants, including quercetin glucosides, dicaffeoylquinic acid derivatives, chlorogenic acid derivatives [5] and triterpene saponins [6,7,8]. Furthermore, four new noroleanane-type triterpene saponins (bigelovii A, bigelovii B, Salbige A and Salbige B) were identified from *S. bigelovii* and *S. herbacea*, and three of them (bigelovii A, Salbige A and Salbige B) exhibited potential antiproliferative activity toward promyelocytic leukemia cancer cells HL-60, human breast cancer cells MCF-7, human liver cancer cells HepG2 and lung cancer cells A549 [6,8]. Additionally, bigelovii A regulated apoptosis of HL-60 cells by the decrease of Bcl-2 and the increase of cleaved caspase-3 [9]. However, its bioactive components in terms of their role in its antiproliferative activities and molecular mechanism have not been entirely conducted.

Herein, we described the separation and structural identification of bigelovii C and four known compounds from *S. bigelovii* Torr. Furthermore, we evaluated the cytotoxic activities of all of the isolated compounds and used MCF7 cells to study the cytotoxic mechanism of bigelovii C.

## 2. Results and Discussion

### 2.1. Structural Elucidation of the Isolated Compounds

**Figure 1:**
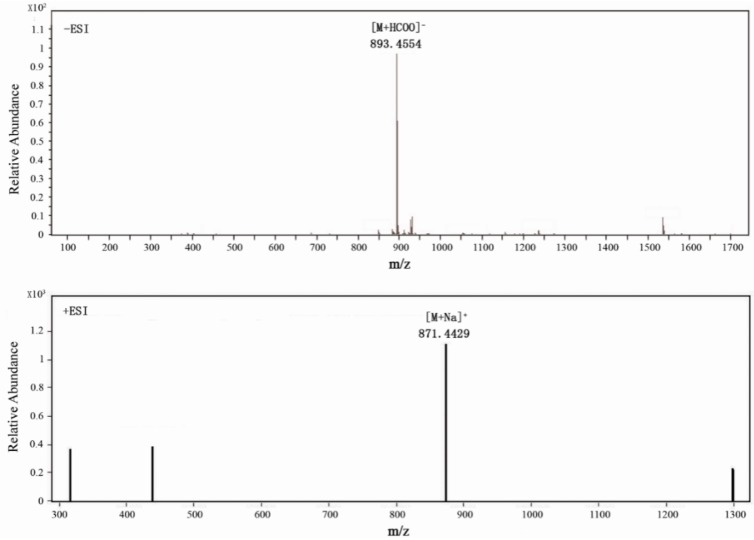
Negative and positive mode of ESI-MS spectra of Compound **1**.

**Figure 2:**
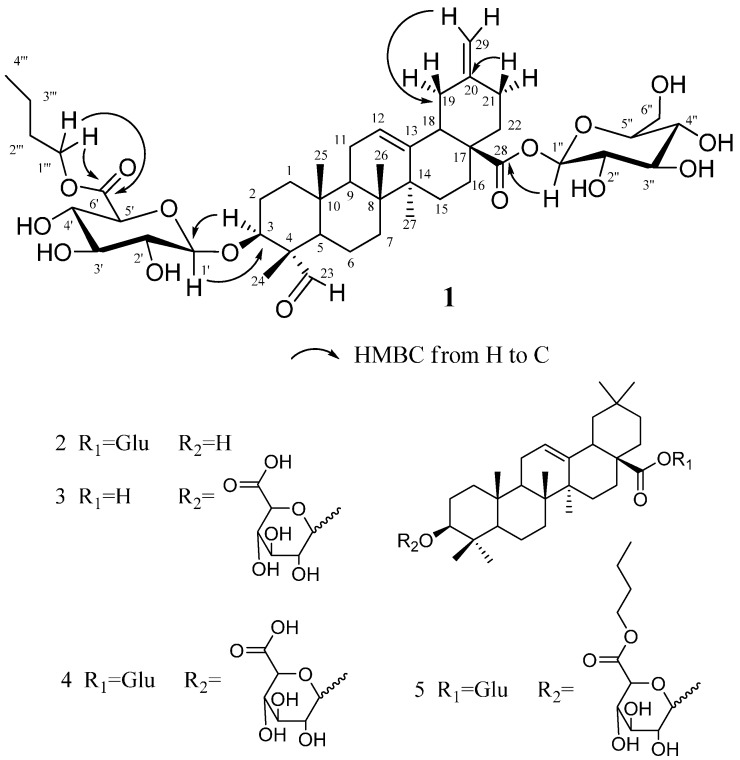
Key HMBC correlations for Compound **1** and the chemical structures of Compounds **2**–**5** isolated from *S. bigelovii* Torr.

Compound **1** was isolated as a colorless needle crystal (MeOH-H_2_O), mp. 159–161 °C and provided positive test results in Molisch’s test and the Liebermann–Burchard test, suggesting that the material was a triterpenoid saponin. TLC, which was sprayed with 10% H_2_SO_4_/EtOH reagent and heated at 105 °C, showed a blue, long placed color that did not fade. As shown in Figure 1, Compound **1** yielded a pseudomolecular [M+HCOO]^−^ peak at *m/z* 893.4554 (calculated 893.4540) and a [M+Na]^+^ peak at *m/z* 871.4429 (calculated 871.4450) by high-resolution mass spectroscopy (HRMS), revealing a molecular formula of C_45_H_68_O_15_ by combining with its 1D-NMR data. Its IR spectrum contained absorption at 3410, 1725, 1650 and 1065 cm^−1^, indicating structures of glycosidic-type, carboxyl and olefinic functionalities. The ^13^C-NMR, ^1^H-NMR (Table 1) and DEPT spectrum collectively revealed that the compound had four tertiary methyl groups at δ 0.83 (3H, s, H-25), 1.01 (3H, s, H-26), 1.20 (3H, s, H-27), 1.28 (3H, s, H-24), one butyl ester at δ 0.76 (3H, t, *J* = 7.4 Hz, H-4'''), 1.33 (2H, m, H-3'''), 1.57 (2H, m, H-2''') and 4.26 (2H, t, *J*=7.35 Hz, H-1'''), one oxy-methine proton signal at δ 4.14 (1H, dd, *J* = 11.6, 4.5 Hz), an aldehyde group proton signal at δ 9.74 (1H, s), two exomethylene protons at δ 4.67 (s, 1H, Ha-29) and 4.74 (s, 1H, Hb-29) and an olefinic proton at δ 5.40 (1H, br s, H-12), as well as two typical sp2 olefinic carbon signals at δ C 123.3 and 143.5. Taken together, these data were indicative of a typical noroleanic-acid-type triterpene. We can see 45 carbon signals from the ^13^C-NMR of Compound **1**, containing 29 carbon signals of the aglycone, 12 carbon signals of the sugar moieties and four carbon signals of a butyl ester group. HMBC analysis of **1** revealed several key correlations, including correlations from H-3 to C-5 and C-24, H-16 to C-18, H-18 to C-20, H-24 to C-3, C-4 and C-23, H-25 to C-1 and C-10, H-26 to C-7 and C-8, H-27 to C-8 and C-15 and H-29 to C-19 and C-21. Furthermore, the signals of the ^1^H and ^13^C-NMR data of **1** with those of akebonoic acid revealed that these compounds possessed similar structural features, except for the signals corresponding to the 23-position. The ^13^C-NMR spectrum of Compound **1** also contained three carbonyl signals at δ 170.1, 175.6 and 206.8, which were assigned to the 6'-glycosyl ester carbonyl, C-28 carbon and 23-aldehyde groups on the basis of the results provided by the HSQC and HMBC spectra, respectively (Figure 2). The butyl group was located at C-6', and the sugar unit identified as a β-glucopyranosyl group was connected at C-3 of the aglycon, which were obtained from the pivotal correlations from H-3 to C-1' and from H-1' to C-3 in HMBC, respectively. The correlations of all of the protons and carbons belonging to the sugar moiety were assigned using a combination of ^1^H-^1^H COSY, HSQC and HMBC analysis. Acid hydrolysis of **1** with 1 N HCl liberated the aglycone and n-butanol, together with d-gluconic acid, which was identified by gas chromatography-mass spectrometry (GC-MS) analysis of the corresponding trimethylsilyl l-cysteine derivative and a direct comparison with an authentic sample of the same material, which had been prepared in the same manner. The results revealed that the acid hydrolysate of the pure Compound **1** contained a d-glucose acid derivative. This result was confirmed by comparing the retention time of this derivative with that of the authentic d-glucose acid derivative, with both samples providing a retention time of 9.52 min. The stereochemistry of Compound **1** was determined by a rotating frame Overhauser effect spectroscopy (ROESY) experiment, which revealed NOE correlations between the following proton pairs: H-5α and δ 9.74 (1H, s) CHO-23; H-24 and H-25; H-3 and δ 9.74 (1H, s) CHO-23; H-3 and δ 4.87 (1H, d) H-1', which confirmed that the position of H-3 and CH_3_-24 was H-3α and CH_3_-24β, respectively. Thus, the structure of **1** was identified as 3-*O*-(6-*O*-butyl ester)-β-d-glucuropyranosyl-23-aldehyde-30-norolean-12, 20 (29)-dien-28-oic acid-28-*O*-β-d-glucopyranoside and named bigelovii C.

**Table 1:** NMR spectroscopic data for Compound **1** in pyridine-*d*_5_ (^1^H: 500 MHz, ^13^C: 125 MHz).

No.	δ_C_, Type	δ_H_ (*J* in Hz)	^1^H-^1^HCOSY	ROESY	HMBC
1	38.1, CH_2_	1.48, 0.93, m	2	2	
2	25.2, CH_2_	2.16, 1.85, m	1, 3	1,3	
3	82.2, CH	4.14, dd (11.6, 4.5)	2	2, 23, 1', 5	23, 1', 5, 24
4	55.4, C				
5	47.9, CH	1.35, dd (7.8, 7.2)	6	3, 6, 23	
6	20.4, CH_2_	1.37, 0.98, m	5, 7	5, 7	
7	32.4, CH_2_	1.40, 1.16, m	6	6	
8	40.2, C				
9	47.9, CH	1.63, m	11	11	10, 8, 11, 25, 26
10	36.1, C				
11	23.6, CH_2_	1.84, m	9, 12	9, 12	
12	123.3, CH	5.40, t (3.5)	11	11	14, 18
13	143.5, C				
14	42.1, C				
15	28.1, CH_2_	2.23, 1.11, m	16	16	
16	23.5, CH_2_	2.14, 2.01, m	15	15	18
17	47.3, C				
18	47.6, CH	3.08, dd (8.7, 4.65)	19	19	20
19	41.6, CH_2_	2.57, m 2.18, t (13.3, 8.7)	18	18	
20	148.4, C				
21	30.1, CH_2_	2.19, 2.03, m	22	22	
22	37.6, CH_2_	1.98, 1.68, m	21	21	20
23	206.8, CH	9.74, s		3, 5	
24	10.4, CH_3_	1.28, s		25	4, 3, 23
25	15.6, CH_3_	0.83, s		24	10, 1
26	17.4, CH_3_	1.01, s			7, 8
27	26.0, CH_3_	1.20, s			15, 8
28	175.6, C				
29	107.2, CH_2_	4.74, 4.67, s			19, 21
3-O-GlcA					
GlcA-1'	105.4, CH	4.87, d (7.75)	2'	2', 3	3
GlcA-2'	75.0, CH	3.94, d (8.5)	3', 1'	3', 1'	1', 3'
GlcA-3'	77.8, CH	4.16, t (8.5, 7.2)	2', 4'	2', 4'	1'
GlcA-4'	72.9, CH	4.43, t (7.2, 9.75)	3', 5'	3', 5'	6'
GlcA-5'	77.3, CH	4.50, d (9.75)	4'	4'	6', 4', 1'
GlcA-6'	170.1, C				
28-*O*-Glc					
Glc-1''	95.8, CH	6.23, d (8.1)	2''	2''	28
Glc-2''	74.0, CH	4.12, d (8.55)	3'', 1''	3'', 1''	1'', 3''
Glc-3''	78.8, CH	4.22, t (8.55, 7.85)	2'', 4''	2'', 4''	4'', 2''
Glc-4''	71.2, CH	4.27, t (7.85, 9.95)	5'', 3''	5'', 3''	5'', 6''
Glc-5''	79.2, CH	3.97, d (9.95)	4'', 6''	4'', 6''	
Glc-6''	62.3, CH_2_	4.41, brd (11.8) 4.33, dd (11.8, 4.55)	5''	5''	
6'-*O*-butyl					
1'''	65.0, CH_2_	4.26, t (7.35)	2'''	2'''	6', 2''', 3'''
2'''	30.8, CH_2_	1.57, m	1''', 3'''	1''', 3'''	4''', 3'''
3'''	19.2, CH_2_	1.33, m	2''', 4'''	2''', 4'''	1''', 4'''
4'''	13.7, CH_3_	0.76, t (7.4)	3'''	3'''	2''', 3'''

### 2.2. Three of the Isolated Compounds, Including Bigelovii C, Had Potent Antiproliferative Activity in Vitro

**Figure 3:**
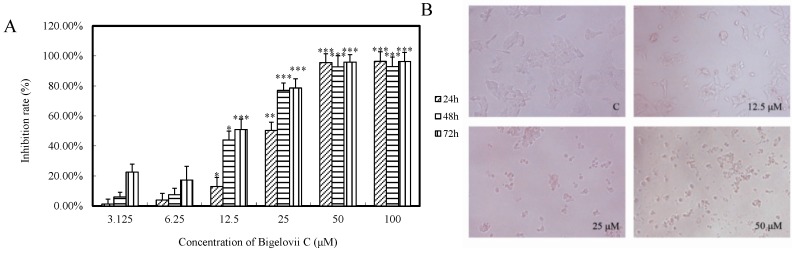
Bigelovii C had potent cytotoxic activity on MCF-7 cells. (**A**) Cells were treated with bigelovii C for 24 h, 48 h and 72 h. Cytotoxicity was evaluated by the MTT assay. * *p* < 0.05, ** *p* < 0.01 and *** *p* < 0.001, compared with the control group. (**B**) Morphological observation of MCF-7 cells treated with 12.5 μM, 25 μM and 50 μM bigelovii C for 24 h under optical microscopic observation (100×).

To evaluate the effects of the five isolated triterpene saponins on cell growth, MTT assays were performed on MCF7, Lovo and LN229 cells, using serial concentrations (0, 3.125, 6.25, 12.5, 25, 50, 100 μM). As shown in Table 2, Compounds **2** and **4** were inactive (IC_50_ > 100 μM), while Compounds **1**, **3** and **5** demonstrated potent cytotoxicity against all three cancer cells, especially MCF7 cells, with IC_50_ values of 12.60 ± 2.49 μM, 13.42 ± 1.22 μM and 3.52 ± 1.19 μM, respectively. MCF7 cells were used to further evaluate the cytotoxic effect of the new saponin, bigelovii C. As shown in Figure 3A, the inhibition rate increased with increasing concentrations of bigelovii C, in a time-dependent fashion. Furthermore, MCF7 cells became round and floated after treatment with 12.5–50 μM bigelovii C for 24 h, while the untreated healthy cells displayed a dissimilar cytoskeleton (Figure 3B).

**Table 2:** *In vitro* cytotoxicity of Compounds **1**–**5** against MCF7, Lovo and LN229 cells (IC_50_, μM) for 72 h.

Compounds	MCF7	Lovo	LN229
**1**	12.6 ± 2.49	21.35 ± 2.55	29.66 ± 6.99
**2**	>100	>100	>100
**3**	13.42 ± 1.22	22.15 ± 3.61	31.18 ± 4.72
**4**	>100	>100	>100
**5**	3.52 ± 1.19	11.45 ± 2.58	10.71 ± 2.26
Cis-platinum	14.73 ± 5.30	51.90 ± 7.15	55.24 ± 7.82

### 2.3. Effects of Bigelovii C on Cell Apoptosis and Mitochondrial Membrane Potential

**Figure 4:**
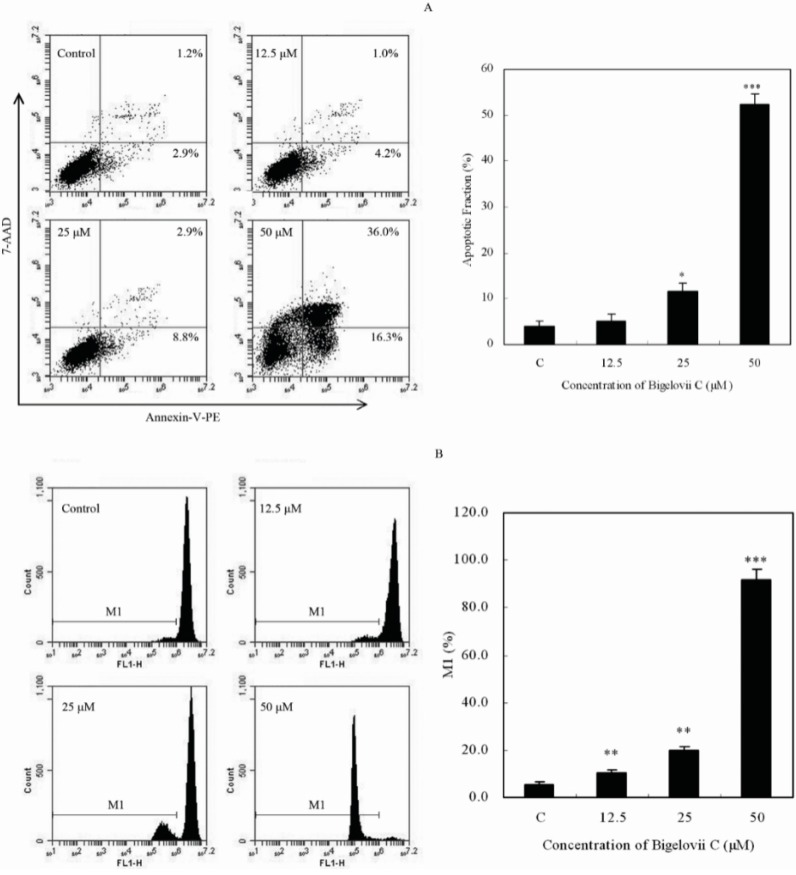
Flow cytometry analysis of MCF7 cell apoptosis (**A**) and mitochondrial membrane potential (**B**). * *p* < 0.05, ** *p* < 0.01 and *** *p* < 0.001, compared with the control group.

To determine whether bigelovii C caused apoptosis, we performed 7-amino-actinomycin (7-AAD) and Annexin V-phycoerythrin (PE) double-staining assays. As shown in Figure 4A, the right quadrants represented apoptotic cells, the percentage of which increased in a concentration-dependent fashion from 4.1% ± 1.1% in the control group to 11.7% ± 1.8% and 52.3% ± 2.4% upon treatment with 25 μM and 50 μM bigelovii C.

Disruption of the mitochondrial membrane potential (MMP) is one of the earliest intracellular events that occurs after the induction of apoptosis. The MMP of bigelovii C-treated MCF7 cells was detected by flow cytometry after staining with Rh123. As shown in Figure 4B, as the concentration of bigelovii C increased, the MMP decreased. These results showed that bigelovii C could induce significant disruption of mitochondrial function.

### 2.4. Effects of Bigelovii C on Apoptosis-Related Protein Levels in MCF7 Cells

**Figure 5:**
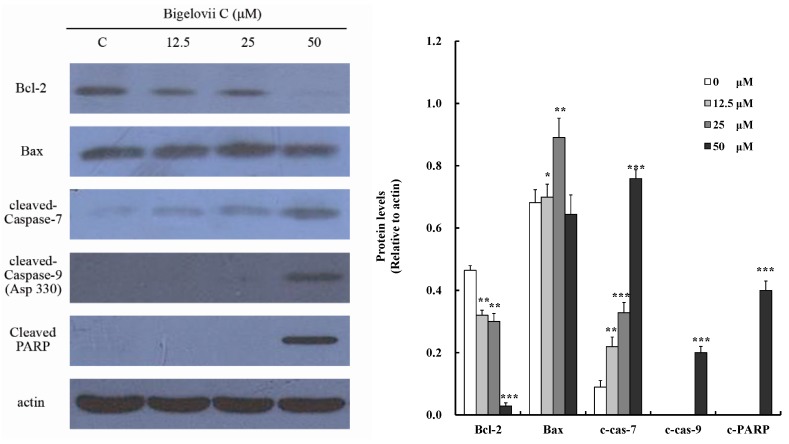
Effects of bigelovii C on the expression of cleaved caspase-7, cleaved caspase-9, cleaved PARP, Bcl-2 and Bax in MCF7 cells. * *p* < 0.05, ** *p* < 0.01 and *** *p* < 0.001, compared with the control group.

To study the mechanism of bigelovii C-regulated apoptosis, apoptosis-related proteins were measured using Western blotting (Figure 5). The expression of Bcl-2, an anti-apoptotic protein, diminished in a dose-dependent fashion, while the expression level of Bax increased after treatment with 12.5 μM and 25 μM bigelovii C, but not 50 μM. Furthermore, cleaved PARP, activated caspase-7 and caspase-9 were observed, while activated caspase-8 was not. Since MCF-7 cells do not express caspase-3, cleaved caspase 3 was not detected [10]. These results indicated that bigelovii C induced apoptosis via regulation of the mitochondrion-mediated apoptosis pathway.

### 2.5. Discussion

Breast cancer is the main reason for death in females worldwide [11]. Plant-derived triterpenoids are regarded as promising candidates to treat breast cancer [12]. In this study, a new noroleanane-type triterpene saponins, bigelovii C (**1**), was isolated from *S. bigelovii* Torr., together with four other known triterpenoids, oleanolic acid 28-*O*-β-d-glucopyranoside (**2**), 3-*O*-β-d-glucuropyranosyl-oleanolic acid (**3**), 3-*O*-β- d-glucuropyranosyl-Oleanolic acid 28-*O*-β-d-glucopyranoside (**4**) and oleanolic acid 3-*O*-β-d-glucuronopyranoside-6-*O*-butyl ester-28-*O*-β-d-glucopyranoside (**5**). As shown in Table 2, saponins **1** and **5**, which have an n-butyl ester on their glucuronic acid moiety, exhibited considerable cytotoxic activity (IC_50_: 12.6–29.66 μM for Compound **1**; 3.52–11.45 μM for Compound **5**) against three tumor cell lines, whereas the compounds with free carboxylic groups, saponins **4** and bigelovii B [6], were inactive (IC_50_ > 100 μM). This result made us speculate that the existence of an n-butyl ester on their glucuronic acid moiety was essential for the cytotoxic activity. Saponin **3**, which has a free carboxylic group at C-28, exhibited moderate cytotoxic activity (IC_50_: 13.42–31.18 μM) against the tumor cell lines, while the compound with a sugar-bonded ester, saponin **4**, was inactive (IC_50_ > 100 μM). Thus, the presence of a free carboxyl at C-28 might be significant for the cytotoxic activity. The above two results were in agreement with those reported previously for similar compounds [6,7,13].

Triterpenoids and their saponins exert antitumor activity through different mechanisms, the most important one of which is the induction of apoptosis [14]. Our data demonstrated that MCF7 cells, following exposure to bigelovii C, exhibited typical apoptotic characteristics, such as externalization of phosphatidylserine on the plasma membrane, in a dose-dependent manner (Figure 4A). There are two identified apoptotic pathways in cells, which are mediated by the death receptor and mitochondria, respectively [15,16]. The mitochondrion transduction pathway is governed by the Bcl-2 protein family [17]. Pro-apoptotic protein Bax transposes to the mitochondrial outer membrane, followed by cytochrome c release and MMP disruption. On the contrary, anti-apoptotic member Bcl-2 preserves mitochondrial integrity to inhibit the process. Mitochondria-dependent death signals recruit and cleave initiator caspase-9, while death receptors result in the activation of caspase-2, -8 or -10. These caspases then activate caspase-3, -6 and -7 [18], which cleaved PARP into p24 and p89, resulting in DNA fragmentation and inducing apoptosis [19,20]. Our investigations showed that bigelovii C disrupted the mitochondrial membrane potential (Figure 4B). Protein analysis demonstrated that bigelovii C reduced Bcl-2 anti-apoptotic protein and activated caspase-9, caspase-7 and PARP in a dose-dependent manner (Figure 5). Therefore, bigelovii C induced apoptosis by the mitochondrion-mediated pathway.

## 3. Experimental Section

### 3.1. General Methods

Melting points were recorded on a XT-4 Boetius micro melting point apparatus (Beijing, China). Optical rotation measurements were conducted by a Perkin-Elmer 341 digital polarimeter (Waltham, MA, USA). A Thermo Nicolet Nexus 870 FT-IR E.S.P. spectrometer (Beijing, China) was used for getting the IR spectrum. The HR-ESI-MS experiment was conducted by using an Agilent 1260 UPLC DAD 6530 QTOF mass spectrometer. NMR spectra were obtained with a Bruker DRX500 NMR spectrometer and pyridine-d5 (C_5_D_5_N) as the solvent at room temperature. Silica gel (200-300 mesh, Qingdao, China), Sephadex LH-20 (Shanghai, China) and C18 silica gel (50 µm, Quebec, Canada) were used for column chromatography. Silica gel G TLC plates (Merck, Darmstadt, Germany) were used for compound detection. The column of Allsphere ODS-2.5 µm (250 × 4.6 mm, Beijing, China) and an Agilent pump 1100 were used for HPLC analysis with the help of the ELSD detector Alltech 500.

### 3.2. Materials and Chemicals

The herb *S. bigelovii* Torr., identified by Gan Yao, were collected from Yancheng in China in June, 2012. C5D5N was obtained from Merck (Darmstadt, Germany). Fetal bovine serum (FBS) and Dulbecco’s Modified Eagle’s Medium (DMEM) were bought from Gibco^TM^ Invitrogen Corp. (Grand Island, NY, USA). Antibiotics, 3-(4,5-dimethylthiazol-2-yl)-2,5-diphenyl tetrazolium bromide (MTT) and RIPA lysis buffer were provided by Sunshine Biotechnology (Nanjing, China). The Annexin V-PE apoptosis kit was supplied by Becton, Dickinson and Company (Franklin Lakes, NJ, USA). Primary antibodies against Bcl-2, Bax, cleaved caspase-3, cleaved caspase-7, cleaved caspase-9, cleaved PARP and β-actin, horseradish peroxidase (HRP)-conjugated secondary antibody and the enhanced chemiluminescence (ECL) kit were bought from Cell Signaling Technology (Boston, MA, USA).

### 3.3. Extraction and Isolation

The dried herb (50 kg) of *S. bigelovii* Torr. was cut and extracted with 80% ethanol (150 L, three times) for 72 hours each time. The resulting ethanol mixture was filtered and evaporated under reduced pressure using a rotary evaporator (BÜCHI R-210) to provide ethanol extract (6.5 kg, 13%). The extract was partitioned into petroleum ether/water (1:1), ethyl acetate/water (1:1) and *n*-BuOH/water (1:1), successively. The combined *n*-BuOH extracts were concentrated under a vacuum to produce the *n*-BuOH extraction product (260.2 g, 0.52%).

The *n*-BuOH extraction product (260.2 g) was chromatographed on normal-phase silica gel column by using CH_2_Cl_2_ containing increasing concentrations of MeOH and H_2_O (95:5:0→90:10:1→80:20:3→70:30:5→60:40:10→50:50:15→30:70:15→0:100:0, v/v/v) as eluents, giving Fractions 1 to 8 with the help of TLC detection.

Fraction 2 (23.6 g) was applied to a Sephadex LH-20 column with an eluent of CH_2_Cl_2_:MeOH (1:1, v/v), yielding five subfractions, labeled as Fractions 2-1 to 2-5, respectively. Then, Fraction 2-3 (1.5 g) was chromatographed via reversed-phase silica gel column using MeOH:H_2_O (20:80 to 100:0, v/v) and further separated through HPLC eluting with MeOH:H_2_O (34:66, v/v) to yield Compound **1** (13.657 mg).

Fraction 3 (7.279 g) was separated into eight subfractions (Fractions 3-1 to 3-8) by using silica gel column with an eluent of EtOAc:MeOH (30:1 to 0:100, v/v). Fraction 3-1 was subjected to a Sephadex LH-20 column with an eluent of CH_2_Cl_2_:MeOH (1:1, v/v), yielding Compound **2** (18.903 mg).

Fraction 7 (27.953 g) was separated by reversed-phase C-18 silica gel column by using an eluent of MeOH:H_2_O (20:80 to 100:0, v/v), providing nine fractions in total (Fractions 7-1 to 7-9). Compound **3** (27.349 mg), Compound **4** (29.376 mg) and Compound **5** (31.952 mg) were respectively obtained by separating Fraction 7-3 (53.930 mg), Fraction 7-5 (37.601 mg) and Fraction 7-4 (65.387 mg) with the help of preparative HPLC, using eluents of MeOH:H_2_O (70:30, v/v), MeOH:H_2_O (45:55, v/v) and MeOH:H_2_O (65:35, v/v).

### 3.4. Cell Culture

MCF-7, Lovo (human colon cancer cell line) and LN229 (human glioblastoma cells) were from the Cell Bank of the Chinese Academy of Science (Shanghai, China). They were cultured in complete DMEM in a 5% CO_2_ atmosphere at 37 °C.

### 3.5. Cytotoxicity Assay

The cytotoxic effects of the five triterpene saponins were evaluated by the MTT assay [21]. Briefly, cells were seeded at 1 × 10^4^ cells per well and treated with serial concentrations of the saponins (0, 3.125, 6.25, 12.5, 25, 50, 100 μM). The treated and control groups each were conducted in sextuplets. Absorbances were recorded on an ELISA reader (Tecan Austria GmbH, Salzburg, Austria) at 570 nm. IC_50_ values were taken as the concentration on which the inhibition rate was 50%.

### 3.6. Annexin-V-PE/7-AAD Analysis

MCF7 cells (4 × 10^5^ cells/well) were seeded and incubated with 12.5 μM, 25 μM and 50 μM bigelovii C for 24 h. Then, they were resuspended in HEPES, stained with 7-AAD and Annexin-V-PE. After incubating in the dark for 15 min, 1 × 10^5^ cells for each sample were analyzed on an Accuri C6 flow cytometer (Becton Dickinson, CA, USA) in 1 h.

### 3.7. Analysis of the Mitochondrial Membrane Potential

The MMP was determined by a cell permeable cationic dye, Rh123 (Sigma, St. Louis, MO, USA). MCF7 cells (4 × 10^5^ cells/well) were seeded and incubated with 12.5 μM, 25 μM and 50 μM bigelovii C for 24 h. Then, the harvested cells were resuspended in PBS buffer, stained with Rh123 (final concentration: 10 μg/mL). After incubating at 37 °C for 0.5 h, the cells were analyzed by flow cytometer.

### 3.8. Western Blotting

Total cell protein extract and Western blotting analysis were conducted according to the previous literature [9] (Guan *et al.*, 2013). Primary antibodies used in this study were Bcl-2, Bax, cleaved caspase-3, cleaved caspase-7, cleaved caspase-8, cleaved caspase-9, cleaved PARP and β-actin.

### 3.9. Statistical Analysis

All experiment data were measured three times. Results are recorded as means ± standard deviation. Statistical analysis compared with untreated control was carried out on the basis of the Student’s *t*-test. If * *p* < 0.05, ** *p* < 0.01 and *** *p* < 0.001, the results were statistically significant.

## 4. Conclusions

In conclusion, five compounds, including a novel oleanane-type 30-nortriterpenoid saponin, bigelovii C, were identified from the n-butanol extract of *S. bigelovii*. Bigelovii C demonstrated potential anticancer activity on MCF7 cells *in vitro* and induced apoptosis through a mitochondrion-dependent pathway. These results suggested that bigelovii C might impart health benefits when consumed and should be regarded as a potential chemopreventative agent for cancer.
